# Maternal perception of malnutrition among infants using verbal and pictorial methods in Kenya

**DOI:** 10.1017/S1368980014001074

**Published:** 2014-05-27

**Authors:** Martha K Mwangome, Gregory Fegan, Andrew M Prentice, James A Berkley

**Affiliations:** 1 KEMRI/Wellcome Trust Research Programme, PO Box 230, Kilifi 80108, Kenya; 2 MRC International Nutrition Group, London School of Hygiene and Tropical Medicine, University of London, London, UK; 3 Centre for Clinical Vaccinology & Tropical Medicine, University of Oxford, Oxford, UK; 4 Medical Research Council Laboratories, MRC Keneba, The Gambia

**Keywords:** Mothers, Undernutrition, Mid-upper arm circumference, Weight-for-age, Infants

## Abstract

**Objective:**

To compare mothers’ perceptions of their own infants’ nutritional status with anthropometric indicators of undernutrition.

**Design:**

A qualitative study and cross-sectional quantitative survey. The qualitative study involved developing tools to assess mother’s perception. Two methods of verbal description and a pictorial scale were developed. The quantitative survey involved measuring maternal perception and comparing it with the anthropometric measures of weight-for-age *Z*-score (WAZ) and mid-upper arm circumference-for-age *Z*-score (MUACZ).

**Setting:**

A rural community setting in Kenya.

**Subjects:**

Seventy-four infants aged between 4 and 6 months, and their mothers, living in rural Kenya were enrolled.

**Results:**

Using verbal description, the positive and negative likelihood ratios were 3·57 (95 % CI 1·44, 9·98) and 0·69 (95 % CI 0·50, 0·96) respectively for MUACZ<−2; and 4·60 (95 % CI 1·60, 13·3) and 0·67 (95 % CI 0·49, 0·92) respectively for WAZ<−2. Using the pictorial scale, the positive and negative likelihood ratios were 8·30 (95 % CI 1·91, 36·3) and 0·69 (95 % CI 0·52, 0·93) respectively for MUACZ<−2; and 4·31 (95 % CI 1·22, 15·0) and 0·78 (95 % CI 0·61, 1·00) respectively for WAZ<−2.

**Conclusions:**

In a rural community, mothers better identify undernutrition in their infants using a pictorial scale than verbal description. However, neither can replace formal anthropometric assessment. Objective anthropometric tools should be validated for identification of severe acute malnutrition among infants aged less than 6 months.

Worldwide, 52 million children aged less than 5 years are wasted (weight-for-height *Z*-score (WHZ) <−2)^(^ ^1^ ^)^. However, because infants below 6 months of age have often been excluded from surveys or rehabilitation programmes, their contribution towards this burden has been undetermined. It was recently estimated that worldwide 8·5 million infants aged less than 6 months are wasted (weight-for-length *Z*-score (WL*Z*) <−2)^(^ ^2^ ^)^. Wasting in infancy and childhood is associated with increased risk of hospital admission^(^ ^3^ ^,^ ^4^ ^)^ and death^(^ ^5^ ^)^. In Kenya, 9·7 % of all infants aged less than 6 months are wasted (WLZ<−2)^(^ ^6^ ^)^. Undernutrition in early infancy may lead to impaired growth and brain development, mental illness^(^ ^7^ ^,^ ^8^ ^)^ and may increase the likelihood of hypertension, CVD, diabetes and chronic thyroid disease in adulthood^(^ ^4^ ^,^ ^9^ ^,^ ^10^ ^)^.

Mothers’ concern and recognition of illness in their children is the first step in seeking health care^(^ ^11^ ^)^. Mothers observe and monitor different aspects of infant growth and form an opinion on the health and nutritional status of their infants^(^ ^12^ ^,^ ^13^ ^)^. These opinions are guided by local norms and influence health-care-seeking decisions and consequently the health of the infant^(^ ^12^ ^)^.

In the developed world, where childhood obesity is a major concern, mothers are reported to misclassify between 30 and 80 % of obese children (BMI≥95th percentile), and between 70 and 98 % of children at risk of overweight (BMI≥85th to <95th percentile), aged 2 to 17 years, as being normal^(^ ^11^ ^–^ ^21^ ^)^. This misperception varies by the child’s sex, maternal weight^(^ ^14^ ^,^ ^19^ ^,^ ^20^ ^)^ and mother’s level of education^(^ ^13^ ^)^. Improving caregivers’ awareness of obesity can enhance early and effective access to interventions^(^ ^17^ ^)^.

In the developing world childhood undernutrition remains prevalent^(^ ^22^ ^,^ ^23^ ^)^, yet the role of maternal perception of nutritional status and effect on the outcome of nutritional interventions is unknown. There is a paucity of studies evaluating maternal perception of undernourished children. Among the few published, considerable variation exists in study design, age and number of participants, geographic location and the mode of assessing maternal perception. Two studies, one among Bangladeshi children aged 3–35 months^(^ ^24^ ^)^ and the other among Colombian children aged less than 4 years^(^ ^25^ ^)^, reported contradictory findings regarding maternal classification of childhood undernutrition. These studies suggest the need for a better understanding of how to measure maternal perception and its contribution to childhood undernutrition. In Kenya, early introduction of complementary food is common and this predisposes infants to the risk of early undernutrition^(^ ^6^ ^)^ and the need for assessment before 6 months of age. The present study was designed to assess mothers’ perception of their infants’ growth and nutritional status and to compare this with anthropometric classification.

## Methods

### Study location and design

A qualitative study and cross-sectional quantitative survey were conducted from September 2011 through April 2012 in Ganze location in Kilifi District, Kenya. A recent survey among community children aged 6–17 months in the district reported the prevalence of wasting (WLZ<−2) as 6·4 %^(^ ^26^ ^)^.

### Study participants

Mothers of infants aged between 4 and 6 months residing in Ganze location were eligible to participate in the study. Mothers were identified using mother–child health clinic records at the Ganze health centre. We set out to recruit every eligible mother in the catchment area of the health centre (using the sub-division geographic boundaries) during the study period rather than select a sample. Registered mothers, community health workers and community members were used to identify and trace unregistered peers.

### Study procedure

We assessed mother’s perception using two approaches, a verbal description and a pictorial scale, and compared these with *Z*-scores for weight-for-age (WAZ) and for mid-upper arm circumference-for-age (MUACZ)^(^ ^27^ ^)^. We used mid-upper arm circumference (MUAC) because of the difficulty of conducting height-based measures where household visits required considerable trekking, and we have recently shown good accuracy and reliability in this age group^(^ ^28^ ^,^ ^29^ ^)^. Although MUACZ were used to facilitate comparison with other *Z*-score indices, in practice, absolute MUAC cut-offs are normally used. Recently, a MUAC cut-off of less than 115 mm was proposed for diagnosing acute malnutrition in infants aged less than 6 months^(^ ^30^ ^)^.

The study was undertaken in three steps as detailed in the following subsections.

#### Pictorial scale development and piloting

A pictorial rating scale was developed to reflect a spectrum of nutritional status in infants aged 4–6 months. Ten infants aged between 4 and 6 months were identified at the mother–child health clinic or paediatric ward at Kilifi District Hospital with MUACZ between −3 and +1. With informed consent, weight, length and MUAC measurement and naked digital photographs were taken. The photographs were converted to ‘pencil-line sketches’ using Adobe Photoshop CS5 software (Adobe Systems Inc., San Jose, CA, USA). The genital area of these images was then obscured. From ten sketches, experts (a medical anthropologist, a nutritionist (M.K.M.) and a paediatrician (J.A.B)) selected five sketches for piloting based on nutritional status, positioning of the infant and clarity. The final five sketches included infants with a MUAC of 90 mm (MUACZ≤−3), 120 mm (MUACZ >−3 to ≤−2), 125 mm (MUACZ >−2 to ≤−1), 135 mm (MUACZ >−1 to ≤0) and 165 mm (MUACZ>0; see Table 1 and online supplementary material, Supplemental Figure 1). The sketches were then printed on A4 paper and laminated to form ‘cards’ used for assessment.

**Table 1:** Questionnaire to assess maternal perception of their infant’s nutritional status, rural Kenya

	Maternal perception question	Possible responses		
Verbal description	**1**. How would you describe the growth and health of your infant?	Very good Good		‘Healthy’
		Not good Poor Very poor		‘Unhealthy’
Pictorial scale	**2**. Organise the sketches in an order of your choice	Perfect order Correct first and last Wrong order		
	**3**. From the sketches, select one that you think:	B – MUACZ≤−3	}	Undernourished
	**a.** Closely resembles the size of your child right now	P – MUACZ>−3 to ≤−2		At risk
	**b.** Closely resembles the ideal size for your child	T – MUACZ>−2 to ≤−1		
	**c.** Closely resembles a child in an alarming health status	N – MUACZ>−1 to ≤0		
		K – MUACZ>0	}	Well nourished

MUACZ, mid-upper arm circumference-for-age *Z*-score.

To assess the acceptability and utility of the scale, four discussion groups each with at least eight caregivers of infants aged 4–6 months visiting Ganze health centre were conducted. During the discussions, participants were encouraged to interact with the sketches and share their opinions regarding the appropriateness, relevance and acceptability of these sketches within their community. Participants were divided into two groups and asked to organise the sketches in any order of their choice. They were encouraged to use local terminologies in describing the selected order of sketches. Field notes were used to capture the contents of these discussions.

#### Questionnaire development and piloting

A structured, interviewer-administered questionnaire with a mixture of open- and closed-ended questions was developed. Maternal characteristics (age, level of education and parity) and infant characteristics (age, sex, vaccination status, recent illness episodes and infant feeding practices) were recorded. Specific questions quantifying maternal perception of their infant’s growth and nutritional status were embedded within the questionnaire, first by verbal description and then by pictorial scale (see Table 1 and online supplementary material, Supplemental Figure 2). Likert-type five-point symmetric scale responses were developed for these questions. For each question, the respondent was presented with a series of potential responses and a numerical value was assigned to each response to enable quantitative statistical analysis.

The questionnaire was piloted using ten in-depth interviews with community mothers of infants aged 4–6 months. Mothers were purposefully selected from the mother–child health clinic at Ganze health centre and were asked for permission to be interviewed. During the interview, questions were asked in the order presented in the questionnaire; responses and any difficulty in flow or comprehension were noted. These were later discussed with the respondent and the study team. The questionnaire was refined iteratively: each interview session used an improved version of the previous questionnaire. Additionally, time required for interview administration was also noted and was used in the consenting process.

#### Data collection

The full questionnaire was administered to mothers who consented to participate in the study. A trained interviewer visited each mother at her home and used the local Giriama language to conduct interviews. Initially, the main questionnaire and verbal description was recorded, and then the pictorial scale was administered. After the interview, the infant’s anthropometric measurements were taken following the procedure described in the UN guide^(^ ^31^ ^)^. Naked weight of the infant was obtained using a Salter scale (model 235–65 (25 kg×100 g); Salter Brecknell, Smethwick, UK) with a precision of 100 g, while MUAC was measured on the left arm of the infant using an insertion tape marker to the nearest millimetre.

### Ethical considerations

The study was approved by the Kenyan National Scientific and Ethical Review Committees (SCC number 1962). For the development of the pictorial scale and participation in the questionnaire survey, written informed consent was sought, while verbal consent was sought during piloting. Only infants of caregivers who consented participated in the study.

### Statistical analysis

Quantitative data were double entered using EpiData software and transferred to the statistical software package STATA 11 for cleaning and analysis. *Z*-scores, including weight-for-age and MUAC-for-age, were computed using the WHO 2006 growth reference standards STATA macro^(^ ^27^ ^)^. Infants were classified as wasted if they had MUACZ<−2 or as underweight if they had WAZ<−2.

Continuous variables were summarized as median and interquartile range (IQR) while categorical variables were presented as frequencies. The association between categorical variables was evaluated using *χ* ^2^ and Fisher exact tests, as appropriate. In order to evaluate the ‘diagnostic’ ability of maternal perception, the likelihood ratio of maternal perception in distinguishing undernutrition among infants was calculated. For that analysis, infants were classified using MUACZ and WAZ <−2 and >–2 as the gold standards. Using maternal perception, all infants classified as ‘unhealthy’ by verbal description or ‘undernourished’ by pictorial scale were considered undernourished while the rest were considered well nourished. Likelihood ratios were chosen for their ability to assess diagnostic tests^(^ ^32^ ^)^ and in our context summarise how more or less likely an undernourished infant is to be classified as such by maternal perception than well-nourished infants^(^ ^32^ ^)^.

## Results

### Results from piloting of study tools

In the discussion groups to pilot the pictorial scale, mothers recognised the sketches as images of infants and noted the different levels of nutritional status. In all groups, participants were able to organise the sketches correctly in a sequential order as informed by the general appearance of the body and its proportionality (e.g. relative sizes of the head and body). We found that there is a local concept of body proportions in relation to health called ‘*mkando*’ in the Kigiriama language (literally ‘moulding’ in English, e.g. a clay model). This concept was used to describe an infant whose head, body and leg dimensions were considered disproportionate.

During the interactive exercises, a poorly nourished child was said to grow poorly ‘*kakulato*’, or be of poor health ‘*kana afya mbizdo*’, or thin ‘*mwambamba/adzaonda*’; while a well-nourished child was said to be growing well ‘*anakulato*’, or of good health ‘*anaafya mbizdo*’, or fat/wobbly ‘*tigwi tigwi*’. It was observed that the local dialect lacked a single word for the English word ‘nutrition’ or phrase ‘nutritional status’; instead words translating to the English words ‘health’, ‘body size/proportion’ and ‘growth’ were used interchangeably to describe the concept of healthy growth. These findings informed the vocabulary, phrasing and order of questions in the final questionnaire.

Overall, these findings suggested that the sketches were acceptable and well comprehended and hence could be used in quantifying maternal perception within this community; and moreover, using suitable vocabulary, that a verbal description of an infant’s nutritional status could be obtained.

### Results from the questionnaire survey

#### Description of study participants

We identified eighty-eight eligible mother–infant dyads; eighty-one from clinical records and seven from local informants and peer-tracing. At the time of survey, eleven of the mothers were away; one mother had died while two mothers could not be traced. Thus data on seventy-four infants (49 % male) and their mothers were available for analysis. The median age of participant mothers was 27 (IQR 23–32) years with a median of four children each (IQR 2–6). Mothers were often illiterate, with thirty-seven (50 %) reported no schooling at all. The median age of the infants was 4·6 (IQR 4·1–5·7) months. The median age for introducing complementary feeds was 3 (IQR 2–4) months. Median MUACZ was –0·72 (IQR −1·14, 0·05) and median WAZ was −0·52 (IQR −1·42, 0·08). Seven (9·5 %) of the infants were wasted (MUACZ<−2), while eight (10·8 %) were underweight (WAZ<−2). Infant nutritional classification by MUACZ varied by mother’s age and years of schooling (*P*<0·05; Table 2).

**Table 2:** Infant and maternal characteristics by infant nutritional status (MUACZ); infants aged 4–6 months, and their mothers, Ganze location in Kilifi District, rural Kenya, September 2011–April 2012

	MUACZ			Total (*n* 74)		
	≤−2	>−2 to ≤−1	>−1			*P* value
	(*n* 7)	(*n* 17)	(*n* 50)	*n*	%	(Fisher’s exact test)
Infant characteristics						
Infant’s sex						
Male	6	9	21	36	49	0·09
Female	1	8	29	38	51	
Infant’s place of birth						
Hospital	3	6	34	43	58	0·09
Home/on the way	4	11	16	31	42	
Infant’s type of breast-feeding						
Exclusive	1	4	6	11	15	0·5
Not exclusive	6	13	44	63	85	
Infant was ill 2 weeks prior						
Yes	4	9	35	48	65	0·4
No	3	8	15	26	35	
Maternal characteristics						
Mother’s age (years)						
<30	1	8	33	42	57	0·02
>30	6	9	17	32	43	
Mother’s years of schooling						
None	2	14	21	37	50	0·02
<4	3	1	10	14	19	
>4	2	2	19	23	31	
Mother’s no. of live children						
<4	3	9	31	43	58	0·6
>4	4	8	19	31	42	

MUACZ, mid-upper arm circumference-for-age *Z*-score.

#### Classification by maternal perception compared with mid-upper arm circumference-for-age and weight for-age Z-scores

In the present study, infants classified as ‘healthy’ by verbal description and ‘well nourished’ by pictorial scale differed significantly in MUACZ and WAZ from those classified as ‘unhealthy’ and ‘undernourished’ (Table 3).

**Table 3:** Maternal perception of their infant’s nutritional status by MUACZ, absolute MUAC and WAZ; infants aged 4–6 months, and their mothers, Ganze location in Kilifi District, rural Kenya, September 2011–April 2012

	MUACZ				*P* value			Likelihood ratio positive (LR+)		Likelihood ratio negative (LR−)	
Maternal perception	≤−2	>−2 to ≤−1	>−1	Total	(Fisher’s exact test)	Sensitivity (%)	Specificity (%)	LR	95 % CI	LR	95 % CI
Verbal description											
Healthy	4	11	45	60							
Unhealthy	3	6	5	14	0·01	38	90	3·57	1·44, 9·98	0·69	0·50, 0·96
Pictorial scale											
Well nourished	0	4	18	22							
At risk	3	9	30	42							
Undernourished	4	4	2	10	0·002	33	96	8·30	1·91, 36·3	0·69	0·52, 0·93
	Absolute MUAC (mm)										
	≤115	>115 to ≤130	>130								
Verbal description											
Healthy	4	28	28	60							
Unhealthy	3	10	1	14	0·014	43	84	2·61	0·95, 7·18	0·68	0·36, 1·31
Pictorial scale											
Well nourished	0	8	14	22							
At risk	3	26	13	42							
Undernourished	4	4	2	10	0·003	57	91	6·38	2·35, 17·3	0·47	0·20, 1·11
	WAZ										
	≤−2	>−2 to ≤−1	>−1								
Verbal description											
Healthy	6	10	44	60							
Unhealthy	2	8	4	14	0·02	39	92	4·60	1·60, 13·3	0·67	0·49, 0·92
Pictorial scale											
Well nourished	0	3	19	22							
At risk	5	11	26	42							
Undernourished	3	4	3	10	0·02	27	94	4·31	1·22, 15·0	0·78	0·61, 1·00

MUACZ, mid-upper arm circumference-for-age *Z*-score; MUAC, mid-upper arm circumference; WAZ, weight-for-age *Z*-score.

Of the seven wasted infants, four (57 %) were incorrectly classified as being healthy by verbal description. Similarly, of the eight underweight infants, six (75 %) were missed by verbal description. The positive and negative likelihood ratios were 3·57 (95 % CI 1·44, 9·98) and 0·69 (95 % CI 0·50, 0·96) respectively for MUACZ<−2; and 4·60 (95 % CI 1·60, 13·3) and 0·67 (95 % CI 0·49, 0·92) respectively for WAZ<−2.

Using the pictorial scale, ten infants were classified by mothers as undernourished, forty-two as being at risk and twenty-two as well nourished (Table 3). Three (43 %) of the seven wasted infants and five (63 %) of the eight underweight infants were misclassified as being ‘at risk’ using the pictorial scale. The positive and negative likelihood ratios were 8·30 (95 % CI 1·91, 36·3) and 0·69 (95 % CI 0·52, 0·93) respectively for MUACZ<−2; and 4·31 (95 % CI 1·22, 15·0) and 0·78 (95 % CI 0·61, 1·00) respectively for WAZ<−2 (Table 3).

#### Mothers’ perception of optimal nutritional status

Mothers were asked to select a sketch that closely resembles the ideal size for their infants (Table 1). In response, sixty-four (88 %) of the mothers selected the images representing the normal and above-normal infant sizes. Further, when asked to identify the sketch representing an infant in an alarming state of health, sixty-three (85 %) of the mothers selected the sketch representing a severely malnourished infant. Maternal responses did not vary by infant’s nutritional status (*P*>0·05).

## Discussion

Our results indicate that among rural, often illiterate mothers, the sensitivity of maternal perception for malnutrition in infancy is low. Mothers were more likely to positively identify undernutrition in their own infants when using the pictorial scale than when using verbal description.

There is a scarcity of published literature evaluating maternal perception of childhood undernutrition in African populations. A study in Columbia agreed with our findings: among 508 children aged 0–4 years, across a spectrum of nutritional status, maternal perception by verbal description poorly predicted child’s nutritional status^(^ ^25^ ^)^. In contrast, in another study involving 339 Bangladeshi children aged 3–35 months, all of whom were recruited from a health facility, the majority of mothers could identify undernutrition in their children by verbal description^(^ ^24^ ^)^. Further, within the Bangladeshi sample, less educated mothers identified wasted (MUAC<125 mm) and underweight (WAZ<70 %) children slightly more often than in our study, with a sensitivity of 59 % and 67 % respectively^(^ ^24^ ^)^. However, the notable differences in age of participants, thresholds used to define wasting and underweight and the prevalence of undernutrition within the sampled populations make it difficult to compare either of these studies with our results.

Because of the risk of death associated with childhood undernutrition^(^ ^5^ ^,^ ^30^ ^)^ it is desirable to have a screening test with high levels of sensitivity. However, subjective measures of nutritional status are likely to be used in contexts of limited preventive and treatment resources. In such circumstances a high specificity, with preferably less than 5 % of false positives, is preferred depending on the interventions on offer^(^ ^33^ ^)^. In our study, high specificity of 96 % was observed for the pictorial scale, suggesting that it may be used in place of MUACZ to identify non-wasted infants. However, this finding needs to be validated on a larger sample of undernourished infants. Overall there is weak evidence for the use of maternal perception in nutritional screening and our results emphasise the use of objective measures of nutritional status for infant assessment. This finding presents a very strong argument for proactive anthropometric screening of infants as visual assessment does not adequately discriminate undernutrition in infants aged below 6 months.

There are no validated tools for assessing maternal perception within our setting. We used two methods to evaluate maternal perception and found the results to differ. One study in the USA reported using a similar approach to assess maternal perception within the context of childhood obesity and found no difference in proportion misclassified by verbal description compared with a pictorial scale^(^ ^12^ ^)^. That study however was conducted among a sample of literate mothers of 2–17-year-old children. Further, the differences in contexts and participant characteristics make the study findings difficult to compare with ours. In order to validate our study’s findings, other studies in similar settings need to be performed.

### Challenges in assessing maternal perception

Our results indicate that depending on the method of assessment applied, mothers’ classification of undernourished infants differed. This difference may be related to several methodological challenges associated with the application of a pictorial scale and verbal description^(^ ^34^ ^)^. In an interview-administered Likert-scale verbal description, the participant is required to listen through the question and the list of possible responses. It is assumed that the participant will comprehend the Likert order before deciding a possible response. This three-stage intellectual process may be challenging for respondents with little or no schooling experience. Alternatively, the interview-administered pictorial scale has the advantage of capturing the attention of the participant^(^ ^35^ ^)^, hence facilitating comprehension of the concept of ordered categories, i.e. the participant can see that the images are of different sizes and make out the right order. In our study, further interaction with the images ensured that the mother considered all the options before selecting a response and hence the increased likelihood of recognizing wasting by the pictorial scale method.

In future, the pictorial scale developed could be used to assess the perception of fathers and grandmothers on infant growth, health and nutritional status. Further, it can be used to facilitate discussions on a series of topics including appropriate infant feeding practices, how to archive ideal infant growth/health, exploring challenges in infant feeding and consequences of infant undernutrition among other topics.

### Strengths and limitations of the study

Using a combined approach provided a complete assessment of maternal perception and made it possible to compare the performance by either of the methods. In the present study all data were collected by one observer, hence increasing the reproducibility and trustworthiness of the study findings. Some limitations to the study include the small proportion (only 10 %) of undernourished infants, which might have increased the probability of not finding a difference in the classification of undernourished infants where there might have been one. However, the study findings were broadly aligned with results reported from a similar study in Bangladesh^(^ ^24^ ^)^ and this increased confidence in the results. Further validation would be achieved by conducting similar evaluation of maternal perception on a larger sample of undernourished infants.

## Conclusion

In the present study, maternal classification by pictorial scale was more accurate than verbal description, but both were associated with under-classification of wasting and underweight in infants. These results confirm the need to perform anthropometric assessment in this age group at a community level. Simple and affordable anthropometric tools such as MUAC should be evaluated for objective nutritional assessment by health workers and possibly mothers of infants within this age group.

## Supplementary material

For supplementary material accompanying this paper visit https://doi.org/10.1017/S1368980014001074. click here to view supplementary material

## References

[1] Black RE, Victora CG, Walker SP et al. (2013) Maternal and child undernutrition and overweight in low-income and middle-income countries. Lancet 382, 427–451. 23746772 10.1016/S0140-6736(13)60937-X

[2] Kerac M, Blencowe H, Grijalva-Eternod C et al. (2011) Prevalence of wasting among under 6-month-old infants in developing countries and implications of new case definitions using WHO growth standards: a secondary data analysis. Arch Dis Child 98, 1008–1013. 10.1136/adc.2010.191882 PMC3195296 21288999

[3] Bejon P, Mohammed S, Mwangi I et al. (2008) Fraction of all hospital admissions and deaths attributable to malnutrition among children in rural Kenya. Am J Clin Nutr 88, 1626–1631. 19064524 10.3945/ajcn.2008.26510 PMC2635111

[4] Gaskin PS, Walker SP, Forrester TE et al. (2000) Early linear growth retardation and later blood pressure. Eur J Clin Nutr 54, 563–567. 10918466 10.1038/sj.ejcn.1601057

[5] Black RE, Allen LH, Bhutta ZA et al. (2008) Maternal and child undernutrition: global and regional exposures and health consequences. Lancet 371, 243–260. 18207566 10.1016/S0140-6736(07)61690-0

[6] Kenya National Bureau of Statistics & ICF Macro (2009) Kenya Demographic and Health Survey 2008–09. Calverton, MD: KNBS and ICF Macro; available at http://www.measuredhs.com/pubs/pdf/FR229/FR229.pdf

[7] Grantham-McGregor S & Baker-Henningham H (2005) Review of the evidence linking protein and energy to mental development. Public Health Nutr 8, 1191–1201. 16277829 10.1079/phn2005805

[8] Victora CG, Adair L, Fall C et al. (2008) Maternal and child undernutrition: consequences for adult health and human capital. Lancet 371, 340–357. 18206223 10.1016/S0140-6736(07)61692-4 PMC2258311

[9] Barker DJ & Clark PM (1997) Fetal undernutrition and disease in later life. Rev Reprod 2, 105–112. 9414472 10.1530/ror.0.0020105

[10] Walker SP, Gaskin P, Powell CA et al. (2001) The effects of birth weight and postnatal linear growth retardation on blood pressure at age 11–12 years. J Epidemiol Community Health 55, 394–398. 11350995 10.1136/jech.55.6.394 PMC1731923

[11] Jeffery AN, Voss LD, Metcalf BS et al. (2005) Parents’ awareness of overweight in themselves and their children: cross sectional study within a cohort (EarlyBird 21). BMJ 330, 23–24. 15567804 10.1136/bmj.38315.451539.F7 PMC539845

[12] Eckstein KC, Mikhail LM, Ariza AJ et al. (2006) Parents’ perceptions of their child’s weight and health. Pediatrics 117, 681–690. 16510647 10.1542/peds.2005-0910

[13] Baughcum AE, Chamberlin LA, Deeks CM et al. (2000) Maternal perceptions of overweight preschool children. Pediatrics 106, 1380–1386. 11099592 10.1542/peds.106.6.1380

[14] Maynard LM, Galuska DA, Blanck HM et al. (2003) Maternal perceptions of weight status of children. Pediatrics 111, 1226–1231. 12728143

[15] Genovesi S, Giussani M, Faini A et al. (2005) Maternal perception of excess weight in children: a survey conducted by paediatricians in the province of Milan. Acta Paediatr 94, 747–752. 16188779 10.1111/j.1651-2227.2005.tb01975.x

[16] Hirschler V, Gonzalez C, Talgham S et al. (2006) Do mothers of overweight Argentinean preschool children perceive them as such? Pediatr Diabetes 7, 201–204. 16911006 10.1111/j.1399-5448.2006.00183.x

[17] Hackie M & Bowles CL (2007) Maternal perception of their overweight children. Public Health Nurs 24, 538–546. 17973731 10.1111/j.1525-1446.2007.00666.x

[18] He M & Evans A (2007) Are parents aware that their children are overweight or obese? Do they care? Can Fam Physician 53, 1493–1499. 17872878 PMC2234629

[19] Mamun AA, McDermott BM, O’Callaghan MJ et al. (2008) Predictors of maternal misclassifications of their offspring’s weight status: a longitudinal study. Int J Obes (Lond) 32, 48–54. 18193064 10.1038/sj.ijo.0803757

[20] Manios Y, Kondaki K, Kourlaba G et al. (2009) Maternal perceptions of their child’s weight status: the GENESIS study. Public Health Nutr 12, 1099–1105. 19105868 10.1017/S1368980008004412

[21] Zonana-Nacach A & Conde-Gaxiola ME (2010) Mothers’ perception of their children’s obesity. Gac Med Mex 146, 165–168. 20957811

[22] Rajaratnam JK, Marcus JR, Flaxman AD et al. (2010) Neonatal, postneonatal, childhood, and under-5 mortality for 187 countries, 1970–2010: a systematic analysis of progress towards Millennium Development Goal 4. Lancet 375, 1988–2008. 20546887 10.1016/S0140-6736(10)60703-9

[23] Victora CG, de Onis M, Hallal PC et al. (2010) Worldwide timing of growth faltering: revisiting implications for interventions. Pediatrics 125, e473–e480. 20156903 10.1542/peds.2009-1519

[24] Roy SK, Rahman MM, Mitra AK et al. (1993) Can mothers identify malnutrition in their children? Health Policy Plan 8, 143–149.

[25] Bertrand WE & Walmus BF (1983) Maternal knowledge, attitudes and practice as predictors of diarrhoeal disease in young children. Int J Epidemiol 12, 205–210. 6874217 10.1093/ije/12.2.205

[26] McOyoo EO (2010) Health, Nutrition and Livelihood Survey of the Greater Kilifi District in the Coastal Province of Kenya. Nairobi: Family Health International.

[27] World Health Organization (2006) The WHO Child Growth Standards. Generva: WHO; available at http://www.who.int/childgrowth/standards/en/

[28] Walker CL, Rudan I, Liu L et al. (2013) Global burden of childhood pneumonia and diarrhoea. Lancet 381, 1405–1416. 23582727 10.1016/S0140-6736(13)60222-6 PMC7159282

[29] Mwangome MK, Fegan G, Mbunya R et al. (2012) Reliability and accuracy of anthropometry performed by community health workers among infants under 6 month in rural Kenya. Trop Med Int Health 7, 622. 10.1111/j.1365-3156.2012.02959.x PMC3963456 22364555

[30] Mwangome MK, Fegan G, Fulford T et al. (2012) Mid-upper arm circumference at age of routine infant vaccination to identify infants at elevated risk of death: a retrospective cohort study in the Gambia. Bull World Health Organ 90, 887–894. 23284194 10.2471/BLT.12.109009 PMC3524961

[31] United Nations (1986) Summary Procedures of, ‘How to Weigh and Measure Children: Assessing the Nutritional Status of Young Children in Household Surveys’. New York: UN Department of Technical Co-operation for Development and Statistics.

[32] Akobeng AK (2007) Understanding diagnostic tests 2: likelihood ratios, pre- and post-test probabilities and their use in clinical practice. Acta Paediatr 96, 487–491. 17306009 10.1111/j.1651-2227.2006.00179.x

[33] Briend A, Maire B, Fontaine O et al. (2011) Mid-upper arm circumference and weight-for-height to identify high-risk malnourished under-five children. Matern Child Nutr 8, 130–133. 21951349 10.1111/j.1740-8709.2011.00340.x PMC6860828

[34] Bernal H, Wooley S & Schensul JJ (1997) The challenge of using Likert-type scales with low-literate ethnic populations. Nurs Res 46, 179–181. 9176508 10.1097/00006199-199705000-00009

[35] Houts PS, Doak CC, Doak LG et al. (2006) The role of pictures in improving health communication: a review of research on attention, comprehension, recall, and adherence. Patient Educ Couns 61, 173–190. 16122896 10.1016/j.pec.2005.05.004

