# Supplementary material for: Maternal perception of malnutrition among infants using verbal and pictorial methods in Kenya
Source: Public Health Nutr. 2014 May 27;18(5):869–76. doi: 10.1017/S1368980014001074 (PMC4396440; doi:10.1017/S1368980014001074)
Supplement: Supplementary file 1 [file S1368980014001074sup.zip › S1368980014001074sup002.pdf]

Date of visit: __/__/____ Study Number: ________________

Village: ____________________________

Information on caretaker

1. Name of caretaker: ___________________
2. DOB caretaker::__/__/____
3. Number of pregnancies: ______________
4. Number of live births: ________________
5. Number of live children:______________
6. Number of children under her care:_________
7. Level of education:

None:=**0** Lower than Std four: =**1** higher than Std four: =**2**
Secondary: =**3** Others: =**4** ☐

Information on infants

8. Name of infant: ___________________
9. DoB of infant:___/__/____
10. Sex: Female: = **1** Male=**2** ☐

11. Type of delivery:
Hospital =**1** Home=**2** On the way=**3**

Describing infant's growth

12. Is the infants still breastfeeding?

Yes =**1** No =**0** ☐

Is the infant taking any of the following drinks or foods?
Water:________ Juice:_________ Cow's milk:_____
Porridge:________ Marshed potatoes Ugali:________

Other foods:________________________________

(**If exclusively breastfeeding=1, if other foods and drinks=0**) ☐

At what age did you start your child on other fluids/foods? ☐

Why did you start your child on other fluids/foods?
____

13. Has your child been unwell in the recent past (2 weeks)

Yes: =**1** No: =**0** ☐

If yes, please describe the illness:
____

14. How many times since birth has the infants visited the health centre for Vaccination alone:______ For illness alone: ______ For vaccination and illness together:______________

    Total number of visits: ☐

15. What infant services have you accessed at your clinic?

Vaccination: ______ Nutrition and health advice: _______
Treatment: _______ Growth monitoring: ___________
Bed nets: _________ None: ________

What other services are available for infants?__________________

Maternal perception of their infant's nutritional status

Fill in Question 7 from the list of choices provided below it

16. How would you describe your infant's growth and health(thrive)?

Very good=**4** Good=**3** Not good=**2** Poor=**1** very poor=**0**

Please look through these sketches. What do you see?

17. Please organize the sketches in an order of your choice

(**Perfect order=2, correct extremes=1, Wrong order=0**) ☐

18. Using sketches presented together but in a random order and allow the mothers time to pick whichever one they think;

i. Closely resembles the size of your child right now: ☐

What did you look for in comparison?

____

ii. Closely resembles the ideal size for your child: ☐

What did you like in this sketch?

____

iii. One that will alarm you to take the child to hospital ☐

What alarmed you about this sketch?

____

19. How would you know if your child is malnourished?

____

Taking and discussing anthropometry

The child's MUAC, Weight and Length to be taken

Weight: _________Length: ___________MUAC: _______________

Do you have any questions or concern from our discussion today?

____
