# Supplementary figures and images for: Maternal perception of malnutrition among infants using verbal and pictorial methods in Kenya
Source: Public Health Nutr. 2014 May 27;18(5):869–76. doi: 10.1017/S1368980014001074 (PMC4396440; doi:10.1017/S1368980014001074)

# Pictorial scale

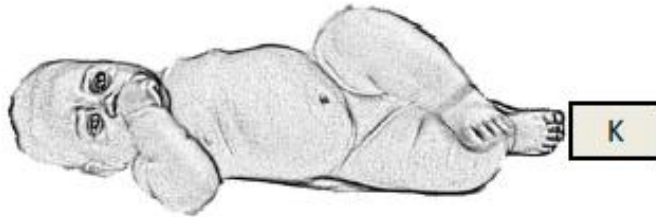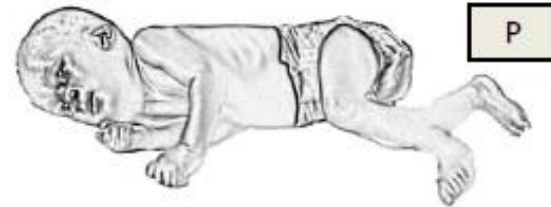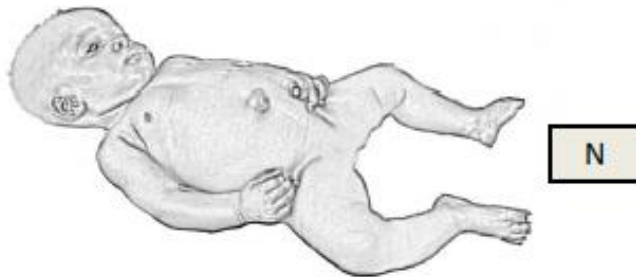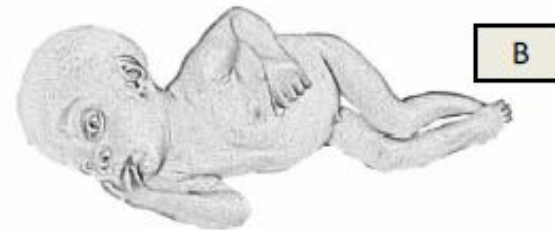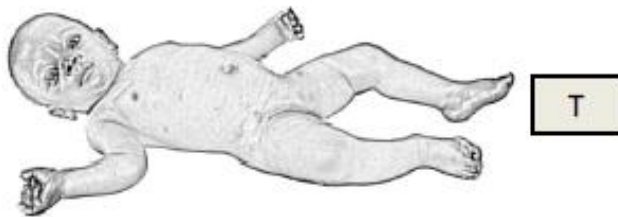

K=  $MUACz \geq 0$

N=  $MUACz > -1$  to  $\leq 0$

T=  $MUACz > 2$  to  $\leq -1$

P=  $MUACz > -3$  to  $\leq -2$

B=  $MUAC \leq -3$

Supplement: Supplementary file 1 [file S1368980014001074sup.zip › S1368980014001074sup001.pdf]
